# The Peroxiredoxin Asp f3 Acts as Redox Sensor in *Aspergillus fumigatus*

**DOI:** 10.3390/genes12050668

**Published:** 2021-04-29

**Authors:** Jana Marie Boysen, Nauman Saeed, Thomas Wolf, Gianni Panagiotou, Falk Hillmann

**Affiliations:** 1 Junior Research Group Evolution of Microbial Interactions, Leibniz-Institute for Natural Product Research and Infection Biology–Hans Knöll Institute (HKI), Beutenbergstr. 11a, 07745 Jena, Germany; Jana.boysen@leibniz-hki.de (J.M.B.); nauman.saeed@leibniz-hki.de (N.S.); 2 Institute of Microbiology, Friedrich Schiller University Jena, 07743 Jena, Germany; 3 Systemsbiology and Bioinformatics, Leibniz-Institute for Natural Product Research and Infection Biology–Hans Knöll Institute, Beutenbergstr. 11a, 07745 Jena, Germany; thomas.wolf@h-s-o.de (T.W.); gianni.panagiotou@leibniz-hki.de (G.P.)

**Keywords:** *Aspergillus fumigatus*, peroxiredoxin Asp f3, AfYap1, transcriptomics, oxidative stress

## Abstract

The human pathogenic fungus *Aspergillus fumigatus* is readily eradicated by the innate immunity of immunocompetent human hosts, but can cause severe infections, such as invasive aspergillosis (IA), in immunocompromised individuals. During infection, the fungal redox homeostasis can be challenged by reactive oxygen species (ROS), either derived from the oxidative burst of innate immune cells or the action of antifungal drugs. The peroxiredoxin Asp f3 was found to be essential to cause IA in mice, but how Asp f3 integrates with fungal redox homeostasis remains unknown. Here, we show that in vivo, Asp f3 acts as a sensor for ROS. While global transcription in fungal hyphae under minimal growth conditions was fully independent of Asp f3, a robust induction of the oxidative stress response required the presence of the peroxiredoxin. Hyphae devoid of Asp f3 failed to activate several redox active genes, like members of the gliotoxin biosynthesis gene cluster and integral members of the Afyap1 regulon, the central activator of the ROS defense machinery in fungi. Upon deletion of the *asp f3* gene Afyap1 displayed significantly reduced nuclear localization during ROS exposure, indicating that Asp f3 can act as an intracellular redox sensor for several target proteins.

## 1. Introduction

The ascomycete *Aspergillus fumigatus* is a ubiquitous fungus which is generally involved in the decomposition of (plant based) biomass, thus playing an important role during carbon and nitrogen recycling [1,2]. With its ability to adapt to a wide temperature range, different oxygen and pH levels and nutritional challenges like low iron levels, *A. fumigatus* thrives in many diverse environments and is also known as the most common airborne fungal pathogen. Infection occurs via the widely distributed asexual conidia which after inhalation, germinate and colonize the lung tissue of immuno-compromised patients such as those suffering from HIV, leukemia or active therapeutic immunosuppression following organ and stem cell transplantation [3,4]. Depending on the underlying condition infections range from allergic bronchopulmonary aspergillosis (ABPA) to often fatal invasive aspergillosis (IA), a disease reaching mortality rates in the range of 30–95% [5,6]. Reasons for high mortality are deficiencies in specific and timely diagnostics as well as the limited availability of effective therapeutic treatment [7]. Hence it is imperative to aim for a better understanding of the pathophysiology of *A. fumigatus*, enabling more targeted approaches towards the development of new therapeutic solutions.

The protein Asp f3 was originally found as a prominent allergen on the surface of fungal conidia [8]. Due to its high abundance and affinity to serum immunoglobulin E (IgE) Asp f3 was also introduced as an auspicious vaccine candidate, protecting Asp f3 immunized mice against invasive pulmonary aspergillosis [9,10]. Recently, we characterized the protein as a dimeric, two-type-cysteine peroxiredoxin Asp f3 which showed peroxidase activity in vitro and protected *A. fumigatus* from external oxidative stress [11]. The protein was furthermore required for virulence in a murine model of pulmonary aspergillosis, and may thus present a promising target for therapeutic applications. Whether its role as a virulence determinant directly relates to its function as a reductive reactive oxygen species (ROS) scavenging enzyme is currently unknown.

Clinical data support an essential role of reactive oxygen species in the defense against fungal infections, as patients suffering from chronic granulomatous disease (CGD) have reduced capability to produce ROS which renders them especially susceptible to *A. fumigatus* infections [7]. The fact that the absence of the major ROS defense activator Yap1 in *A. fumigatus* causes hypersensitivity to ROS, but at the same time does not attenuate its virulence, makes it unlikely that there is a direct correlation between efficient ROS scavenging and virulence. However, ROS may still impact survival of *A. fumigatus* in the host. Only recently host derived ROS were observed to induce the programmed cell death in conidia of the fungus after ingestion by innate immune phagocytes [12]. Here we examine the role of Asp f3 in *A. fumigatus* in the response towards ROS. We show that the absence of Asp f3 does not affect the fungal transcriptome under unstressed conditions but leads to a significant shift in gene expression upon challenge with oxidative stress. Surprisingly, ROS exposure to cells lacking Asp f3 did not activate the AfYap1 regulon, suggesting that Asp f3 acts as an essential redox switch to launch a potent defense against ROS or ROS mediated damages.

## 2. Materials and Methods

### 2.1. A. fumigatus Strain and Culture Conditions

All strains and plasmids used in the study are listed in Table 1. The *asp f3* deletion and complemented strains were generated as described by Hillmann et al. [11]. *A. fumigatus* was cultured on/in *Aspergillus* minimal medium (AMM) with 1% Glucose as carbon source and 20 mM NaNO_3_ as nitrogen source by inoculation with 10^5^ conidia if not otherwise noted [13]. Liquid cultures were kept shaking at 180 rpm at 37 °C for 24 h. Conidia were harvested with 0.1% (*v*/*v*) Tween 80 from AMM-agar plates cultivated at 37 °C for 96 h. Mutant-phenotypes were selected by either 250 µg/mL hygromycin B (Invivogen, Toulouse, France) or pyrithiamine (0.1 mg/mL, Sigma-Aldrich, Taufkirchen, Germany), depending on the resistance marker used in transformation [14]. Conidia were counted by a CASY^®^ Modell TT (OLS OMNI Life Science, Bremen, Germany). For long–time storage, conidia were mixed with glycerol at 20% (*v*/*v*) and frozen at −80 °C.

### 2.2. Construction of Fluorescent Reporter Strains 

For the generation of a *VENUS*-Fusion protein expression strain, the gene sequence of *Afyap1* was cloned into plasmid pGpdA-Afyap1-VENUS containing a constitutive *gpdA*-promoter and *VENUS* gene with a *nos* terminator (Supplementary Figure S1). The target gene (*Afyap1*) was introduced as a N-terminal-fusion to *VENUS* via CPEC and further paired with the *PtrA* resistance cassette which confers resistance to the antimetabolite pyrithiamine [16]. For a list of Primers see Supplementary Table S1. Plasmids were amplified in *E. coli* DH5α, linear fragments for transformation were amplified via PCR (Phusion Flash Polymerase, Thermo Fisher Scientific, Bremen, Germany) and transformed into *A. fumigatus* D141 and Δ*asp f3* via protoplast formation [17,18]. Mutants were confirmed by diagnostic PCR and a detectable *VENUS*-signal during fluorescence microscopy.

### 2.3. Isolation of Chromosomal DNA

Fungal strains were cultivated for 16 h at 37 °C at 180 rpm in Sabouraud 2% Glucose Bouillon (Carl Roth, Karlsruhe, Germany). The mycelium was harvested through miracloth, washed thoroughly with H_2_O, dried and frozen with liquid nitrogen. Frozen mycelium was then ground to a fine powder in a mortar and stored at −20 °C until further use. Isolation of chromosomal DNA was carried out as described previously [19].

### 2.4. Oxidative Stress Experiments 

Reactive oxygen species were produced either by addition of H_2_O_2_ or directly in vivo we with the xanthine oxidase enzymatic system generating a mixture of H_2_O_2_ and O_2_^−^ as previously described [11]. Prior to treatment 10^5^ conidia were grown in liquid Czapek Dox medium (BD, Franklin Lakes, NJ, USA) in 6-well tissue culture plates (VWR International, Leuven, Belgium) in a final volume of 3 mL and cultured for 48 h until a thin layer of mycelium was formed. A sub-lethal concentration (for Δ*asp f3*) of 150 µM xanthine was supplied. The addition of 100 µg/mL (0.2 units/mL) xanthine oxidase (Sigma-Aldrich, Taufkirchen, Germany) started the reaction. For the transcriptome, analysis reaction was stopped after 15 min and samples were harvested, frozen with liquid nitrogen and stored at −80 °C until further use. All data analyzed originated from three biological replicates.

### 2.5. RNA Isolation 

Total RNA was isolated from ROS treated and untreated mycelia of the wild type and the Δ*asp f3* strain using the RNeasy Plant Mini Kit (Qiagen, Hilden, Germany). Frozen mycelium was transferred to tubes containing glass beads (Sigma-Aldrich, Taufkirchen, Germany), after addition of resuspension buffer cells were disrupted by mechanical force applied via FastPrep (MP Biomedicals, Irvine, CA, USA) for 60 s at high-speed setting (6.0). Further processing was conducted according to the manufacturer’s protocol. Extracted RNA was stored at −80 °C. RNA concentration was determined by NanoDrop ND1000 Spectrophotometer (NanoDrop Technologies Inc., Wilmington, DE, USA).

### 2.6. Analysis of Transcriptome Data

The preparation of cDNA libraries from total RNA and the sequencing was performed by GATC Biotech (GATC Biotech, Konstanz, Germany). According to GATC Protocol samples were enriched for mRNA by isolation of poly(A)+ mRNA, mRNA was fragmented and cDNA synthesis was performed to generate strand specific cDNA libraries. From these libraries 1 × 50 bp single end reads were sequenced with the Genome Sequencer Illumina HiSeq (HiSeq 4000 50bp SR) (Illumina, San Diego, CA, USA). FastQC [20] and Trimmomatic v0.32 [21] were used for quality control and trimming of library adaptors. Mapping of reads was achieved with HiSat2 [22] against the reference genome of *A. fumigatus A293*. The normalized number of reads were analyzed with EdgeR, Limma, DESeq, DESeq2 [23,24,25,26] and genes were considered as differentially expressed gene (DEGs) when the differences in the number of reads were statistically significant according to one or more of these tests.

### 2.7. Gene Expression Analysis by qRT-PCR

*A. fumigatus* WT and Δ*asp f3* conidia (1 × 10^9^) were grown in 3 mL AMM and CD at 37 °C and 180 rpm for 6 h to induce swelling. After 6 h, swollen conidia were treated with 0.1 M H_2_O_2_ for 15 min. Swollen conidia were harvested from triplicate samples at 0 and 15 min after the addition of 0.1 M H_2_O_2_ by centrifugation at 800 g and 4 °C for 5 min. Swollen conidia were subsequently lysed with glass beads in the FastPrep (MP Biomedicals, Irvine, CA, USA) for 60 s at 13,000 rpm and processed for total RNA isolation using a Qiagen RNeasy plant mini kit (Qiagen, Hilden, Germany), according to manufacturer’s protocol. Extracted RNA was then stored at −20 °C. Concentration of each sample was determined with NanoDrop ND1000 spectrophotometer (NanoDrop Technologies Inc., Wilmington, DE, USA).

RNA was treated with RQ1 RNase-free DNase (Promega, Walldorf, Germany) and transcribed into cDNA (RevertAid First Strand cDNA Synthesis Kit, Thermo Fischer Scientific, Germany), according to the manufacturers protocol. Quantitative real time polymerase chain reaction (qRT-PCR) was performed using the cDNA as a template. The ΔCT method was used to analyze the relative expression of the target genes, normalized to the constitutively expressed *tubA* gene encoding tubulin A. Al primers are listed in Supplementary Table S1. The reactions were carried out in a total volume of 20 μL on Quantstudio3 system (Thermo Fisher Scientific, Bremen, Germany).

### 2.8. DAPI Staining and Fluorescence Microscopy

A total of 10^5^ conidia were incubated at 37 °C for 10 h in 300 μL of CD in ibidi^®^ μ-slide (ibidi, Gräfelfing, Germany) until germination. The nuclei were then stained with NucBlue^™^ Live ReadyProbes^™^ Reagent (ThermoFisher, Dreieich, Germany) according to manufacturer’s guidelines. Afterwards, 2 mM H_2_O_2_ was added and after 30 min the samples were subsequently analyzed under the microscope. Fluorescent stain and proteins were excited with 408 nm and 488 nm, respectively, to analyze the localization of AfYap1^VENUS^ in both WT and Δ*asp f3* strains using a Zeiss Axio Observer Spinning Disk Confocal Microscope (ZEISS, Jena, Germany) using ZEN software (Version 2.6). Microscopic images were evenly processed and analyzed with ImageJ software [27].

### 2.9. Co-Localization Analysis

For the co-localization analysis of AfYap1^VENUS^ and DAPI, the Coloc2 plugin of ImageJ was used to calculate the Pearson’s correlation coefficient to identify the intensity correlation of fluorescence signals. GraphPad9 Prism software was used to plot the graph and calculate the *p*-value. Error bars represent ± standard deviation from at least 3 images.

### 2.10. Preparation of Protein Extracts and Catalase Activity Measurements

Crude protein extracts were prepared from *A. fumigatus* swollen conidia incubated for 6 h in CD medium and treated with 2 mM of H_2_O_2_ for 45 min. Conidia were harvested by centrifugation and washed thoroughly with PBS. Conidia were re-suspended in assay buffer (Abcam, Cambridge, UK) and disrupted by FastPrep treatment, repeated mixing and sonication for 10 min to enhance the solubilization of proteins. Protein concentration was determined by the Bradford assay [28] and spectrophotometric measurements (UV mini 1240, Shimadzu, Kyoto, Japan). Catalase activity was determined using the catalase activity assay kit (cat. No. ab83464 Catalase Activity Assay Kit, Abcam, Cambridge, UK) according to the manufacturer’s instructions and fluorometric measurements in a fluorescence plate reader (Tecan, Männedorf, Switzerland) at excitation and emission wavelengths of 535 and 587 nm, respectively.

### 2.11. In-Gel Catalase Activity Assay

*A. fumigatus* conidia of different strains were inoculated in CD medium and grown for 20 h at 37 °C prior to a 5 mM H_2_O_2_ treatment for 45 min. The mycelium was harvested, frozen in liquid nitrogen and ground to a fine powder. Isolation of native protein occurred according to Lessing et. al. (2007) [29]. Again, protein concentration was measurured via Bradford assay [28] and spectrophotometric measurements (UV mini 1240, Shimadzu, Kyōto, Japan). A total of 30 µg of protein was loaded on an 8–16% polyacrylamide (wt/vo) Tris-Glycine Gel (Invitrogen™ Novex™ WedgeWell™, Art. Nr.: 15486814 ). The runtime was for 4 h at 60 V at 4 °C. Catalase activity in the gel was determined with the method described by Goldberg and Hochman [30]. A total of 1 µg catalase from bovine liver (C1345, 2000–5000 units/mg protein, Sigma-Aldrich, Taufkirchen, Germany) was used as a positive control. 

### 2.12. Functional Annotation of Transcriptome Data

Enrichment analyses of genes were carried out with the FungiFun2 package [31]. Default settings were used to enrich genes according to GO and FunCat categories. Hits were deemed significant with a *p*-values < 0.01. Enrichment was carried out for the Go-terms “biological process” and “molecular function”. Additional analyses were performed with the AspGD Gene Ontology Term Finder (http://www.aspergillusgenome.org/cgi-bin/GO/goTermFinder, accessed on 28 February 2021) [32].

## 3. Results

### 3.1. Global Transcriptome Analysis Reveals a ROS Specific Function of Asp f3 

**Figure 1:**
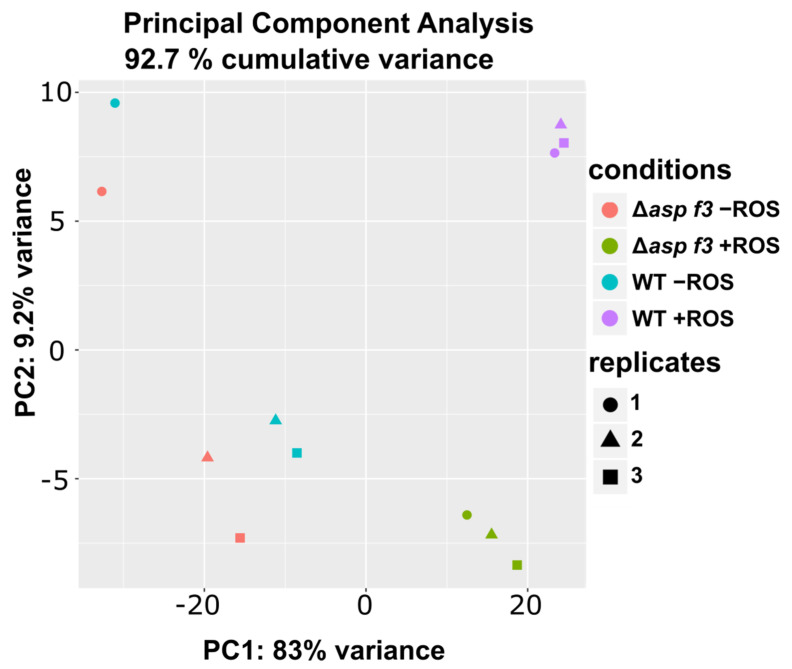
Principal component analysis of transcriptome data from wild type *Aspergillus fumigatus* (WT) and a strain lacking the *asp f3* gene (Δ*asp f3*) grown with (+ROS) or without (−ROS) reactive oxygen species (ROS). The strains, treatments and biological replicates are represented by different colours and symbols, respectively.

To understand the protective role of Asp f3 during ROS exposure we monitored global transcription in wild type hyphae of *A. fumigatus* (WT) and the ROS sensitive deletion mutant Δ*asp f3.* Both strains were first grown in minimal medium and either left untreated (−ROS) or exposed to H_2_O_2_ and O_2_^−^ (+ROS), therefore ROS were generated in vivo by the xanthine oxidase enzymatic system for 15 min in biological triplicates. A principal component analysis of the fungal transcriptomes demonstrated a comparatively low cumulative variance in untreated samples, indicating that the lack of Asp f3, despite its abundance as protein, did not significantly impact transcription under ambient growth conditions of fungal hyphae in minimal medium (Figure 1). These results corresponded to a widely indistinguishable phenotype under a wide range of growth conditions with various carbon sources (Supplementary Figure S2).

In fact, in the absence of ROS, WT and Δ*asp f3* showed an exceptionally high similarity in their genome expression and only seven genes were found to be differentially expressed genes (DEGs). The only DEG commonly identified by all four methods was *asp f3* which remained undetected in the mutant strain, as expected. This indicated that Asp f3 plays a minor role in the absence of ROS and allowed a direct comparison of the ROS-treated samples without a background of genes that directly respond to the absence of Asp f3.

**Figure 2:**
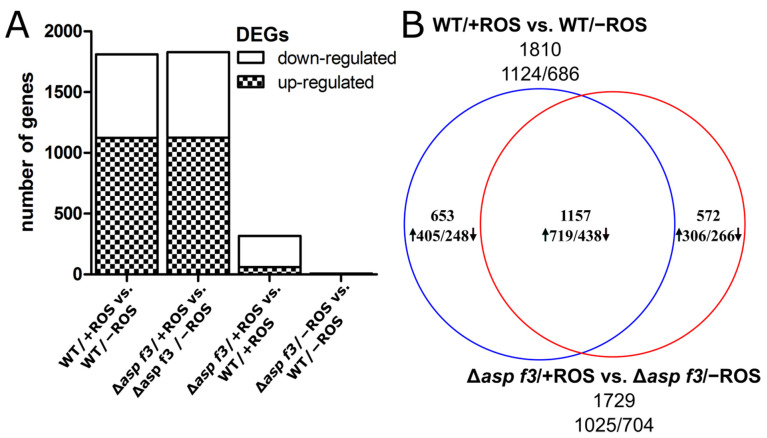
Reactive oxygen species dependent gene expression in *Aspergillus fumigatus* wild type (WT) and hyphae lacking Asp f3 (Δ*asp f3*). (**A**): Comparison of the total number of differentially expressed genes (DEGs) and the direction of their regulation between strains and conditions. (**B**): Venn-diagram of DEGs in the transcriptomes of the WT and Δ*asp f3* during ROS exposure. Given numbers represent the total number of DEGs. Arrows pointing up- or downwards indicate the direction of regulation.

ROS exposure induced a shift in the global transcriptome with nearly identical numbers of DEGs for both strains. For the wild type and Δ*asp f3* mutant we identified 1810 and 1729 ROS dependent DEGs, respectively. The numbers of up- and downregulated genes are likewise similar, identifying 1124 up- and 686 downregulated genes for the WT and 1025 up- and 704 down-regulated genes in Δ*asp f3* (Figure 2A). However, when the DEGs were compared between the two strains, we found only about two thirds of them in congruency. This proportion was comparable for up- as well as for the down-regulated genes (Figure 2B).

### 3.2. Transcriptional Induction of the ROS Defense Requires Asp f3

**Figure 3:**
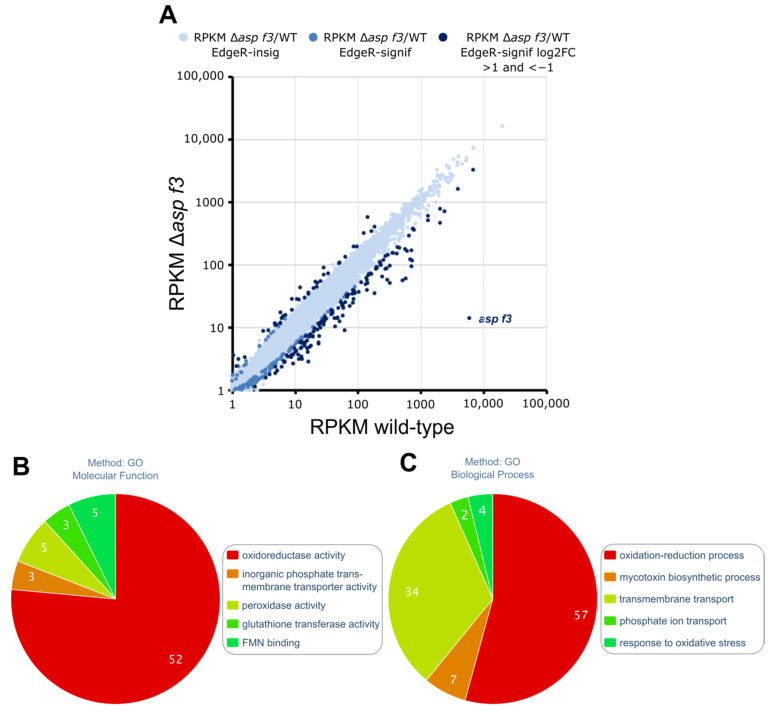
Global transcription in wild type and an *asp f3* deletion mutant of *Aspergillus fumigatus* after treatment with ROS. (**A**): Differential gene expression displayed as RPKM values of single genes in the *asp f3* deletion mutant (Δ*asp f3*) and the wild type (WT) during oxidative stress. Differences in gene expression were defined as either insignificant (bright blue) or significant (blue) according to EdgeR. Genes with log2 fold differences >1 and <−1 are highlighted in dark blue. (**B**,**C**): Gene ontology enrichment according to *Molecular function* (**B**) and *Biological process* (**C**) of genes specifically down-regulated in Δ*asp f3* in response to ROS.

Although the number of ROS induced DEGs was similar in hyphae of the wild type and Δ*asp f3,* 653 genes were differentially expressed only in the wild type, indicating that Asp f3 was required for gene expression under oxidative stress and Δ*asp f3* seems to react differently when challenged with ROS. To get an overview of the effects triggered by loss of the peroxiredoxin we plotted the expression of all genes in WT and Δ*asp f3* under ROS exposure (Figure 3A).

This direct comparison of the treated samples showed 319 DEGs of which 60 were higher expressed in hyphae of the mutant when compared to those of the wild type. The remaining 259 genes showed lower expression in the mutant during ROS exposure. The lowest was the *asp f3* gene itself with RPKM values of 14 and approximately 5900 in hyphae of the mutant and the wild type, respectively.

**Figure 4:**
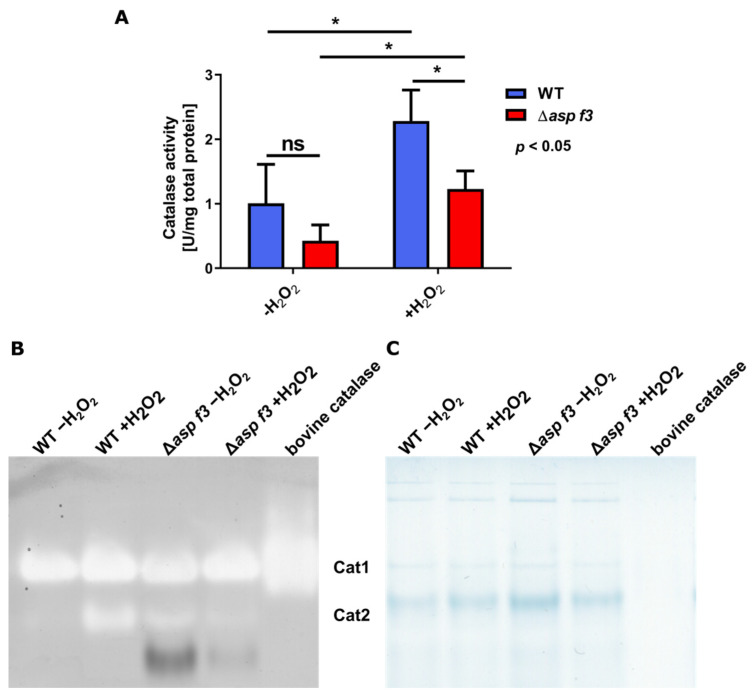
Catalase activity in hyphae of the wild type and the *asp f3* deletion mutant of *Aspergillus fumigatus.* (**A**): Catalase activity was measured in total protein isolated from swollen spores 30 min after stress treatment with either 0 mM (−H_2_O_2_) or 2 mM (+H_2_O_2_) H_2_O_2_. (**B**): Catalase activity staining was performed according to the method of Goldberg and Hochman [30]. Cultures of wild type and Δ*asp f3* were grown for 20 h and treated with 5 mM H_2_O_2_. Negative staining shows catalase activity of Cat1 and Cat2 as described previously [29,33] (**C**): Loading control stained with Coomassie. *: *p* < 0.05

Of the 259 genes that were specifically downregulated in Δ*asp f3* under ROS, 53 genes were not affected by ROS in the wild type. The remaining 205 of these DEGs were up-regulated after ROS exposure of the wild type. A gene ontology analysis for *molecular functions* of the group of genes that lacked ROS dependent expression in the absence of the peroxiredoxin identified primarily oxidoreductase activity, inorganic phosphate transmembrane transporter activity, peroxidase activity and FMN binding as significantly enriched categories (Figure 3B, Supplementary Table S2). *Biological processes* that showed attenuated expression specific to the absence of Asp f3 included categories such as phosphate ion transport, transmembrane transport and mycotoxin biosynthetic processes. The latter was in principle limited to gliotoxin biosynthesis (Figure 3C, Supplementary Table S3). Indeed, a closer look at the genes of the gliotoxin cluster identified 7 of 12 genes as downregulated, including *gliP*, the gene for the non-ribosomal peptide synthetase. The largest enriched biological processes were oxidation-reduction processes and transmembrane transport. More specifically, the transmembrane transport of phosphate ions and the specific response to oxidative stress was deregulated in the absence of the peroxiredoxin. To analyze whether Asp f3 is required for the activation of ROS defense, catalase activity was determined in total protein extracts from both strains before and after a treatment with H_2_O_2_ by indirect measurement of catalase activity (Figure 4A). Untreated hyphae (−H_2_O_2_) revealed insignificant differences in catalase activity. Exposure of H_2_O_2_ to the swollen spores increased catalase activity in both WT and Δ*asp f3*, but ROS dependent upregulation in Δ*asp f3* led to significantly lower activity then in hyphae of the wild type. To determine which catalases were active in the samples both strains were grown for 20 h and challenged with H_2_O_2_ for 45 min. Proteins were extracted and loaded in equal amounts (30 µg) on a native polyacrylamide gel to perform an in-gel catalase activity assay (Figure 4B,C). Catalase activity was visualized by a negative staining and shows a strong induction of Cat2 in the wild type after challenge with H_2_O_2_. In contrast to the wild type, Cat2 activity was detectable in the unchallenged sample of Δ*asp f3*. However, exposure to H_2_O_2_ did not lead to an induction of Cat2 activity. Activity of the spore borne catalase CatA was not observed.

### 3.3. Full ROS Dependent Activation of Several Afyap1 Target Genes Requires Asp f3 

**Figure 5:**
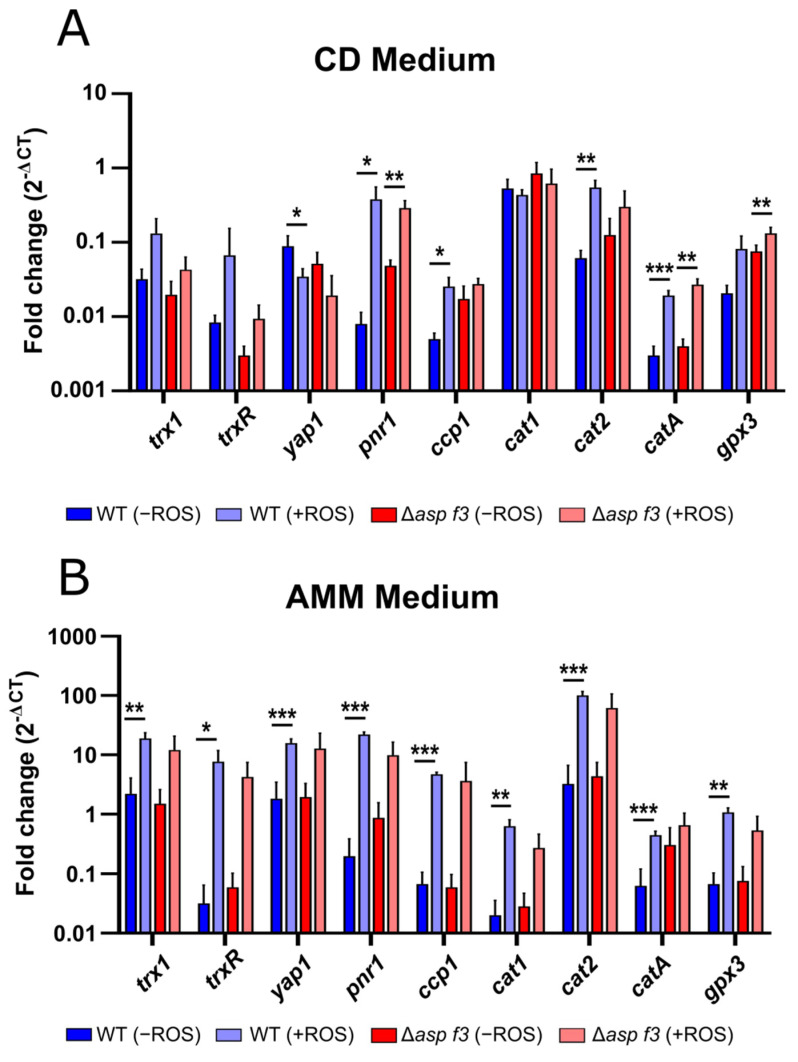
Gene expression of ROS defense genes in wild type (WT) and *aspf3*-deleted (Δ*asp f3*) hyphae of *Aspergillus fumigatus* in Czapek Dox (**A**) and *Aspergillus* minimal medium (**B**) in the absence (−ROS) or presence (+ROS) oxidative stress. Data from qRT-PCR are displayed as logFC normalized to the housekeeping gene *tubA* and represent the mean and SD of three biological replicates. For statistical analysis, Student’s t-test with *: *p* < 0.1; **: *p* < 0.05; ***: *p* < 0.01 was used.

Several genes with a major role in the redox-homeostasis and the defense against oxidative stress were upregulated in the wild type (Table 2), among them three confirmed targets of the major activator AfYap1 [29]: the bifunctional catalase-peroxidase (*cat2*, AFUA_8g01670), the Cytochrome c peroxidase (*ccp1*, AFUA_4G09110), and the p-Nitroreductase family protein (*pnr1*, AFUA_5g09910). The latter is not only known to be strongly induced by diverse environmental stresses such as superoxide stress, osmotic stress and heat stress but is also expressed when the fungus is exposed to neutrophils [34,35]. However, other putative AfYap1-targets suggested by Lessing and colleagues [29], such as the mitochondrial peroxiredoxin (Prx1) or methionine synthase, were not significantly affected in the transcriptome data set of the wild type or the mutant. The expression of the putative Afyap1 targets, the *yap1* itself, and several other ROS defense genes was analyzed by qRT-PCR in two different conditions (Figure 5). Expression of ROS defense genes in Czapek-Dox (CD) medium (Figure 5A), which was also used to generate the transcriptome data, was approximately an order of magnitude lower than in *Aspergillus* minimal medium (AMM) which was supplemented with added trace metals as a major difference (Figure 5B). For both wild type and Δ*asp f3* the expression of the *Afyap1* gene was comparably lower in CD than in AMM even without the addition of ROS stress. For both strains the transcript levels were also not significantly affected by the addition of ROS in CD, while a significant induction of the regulator gene *Afyap1* was detected for both strains in AMM. For several other ROS defense genes, including the putative Afyap1 targets *ccp1*, *cat2*, and *pnr1*, ROS dependent activation was lower in hyphae of the *asp f3* deletion mutant. This lack of activation was also more pronounced in the trace metal free CD medium. ROS mediated induction of *catA* and *gpx3* were detected for both strains in both media. 

### 3.4. Asp f3 Is Required for Afyap1 Activation and Nuclear Localization 

**Figure 6:**
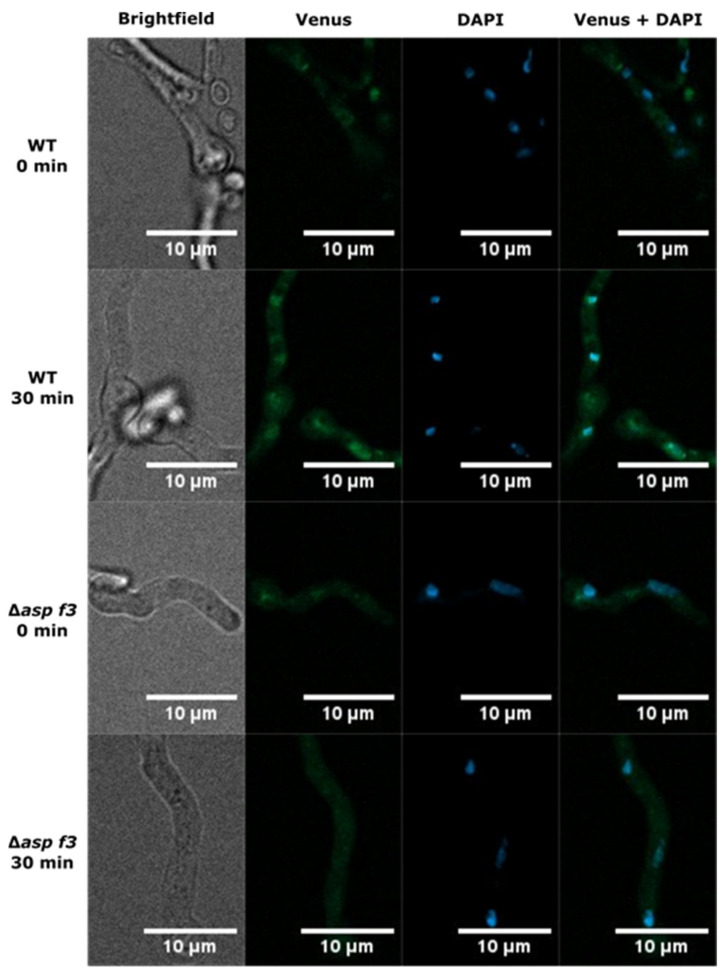
Subcellular localization of AfYap1^VENUS^. *Aspergillus fumigatus* conidia were incubated in Czapec Dox for 10 h until germination. Both strains were challenged with 2 mM H_2_O_2_ for 30 min before microscopy. The VENUS-tag shows a green fluorescent signal for the target protein AfYap1, nuclei were stained with NucBlue™ Live ReadyProbes™.

**Figure 7:**
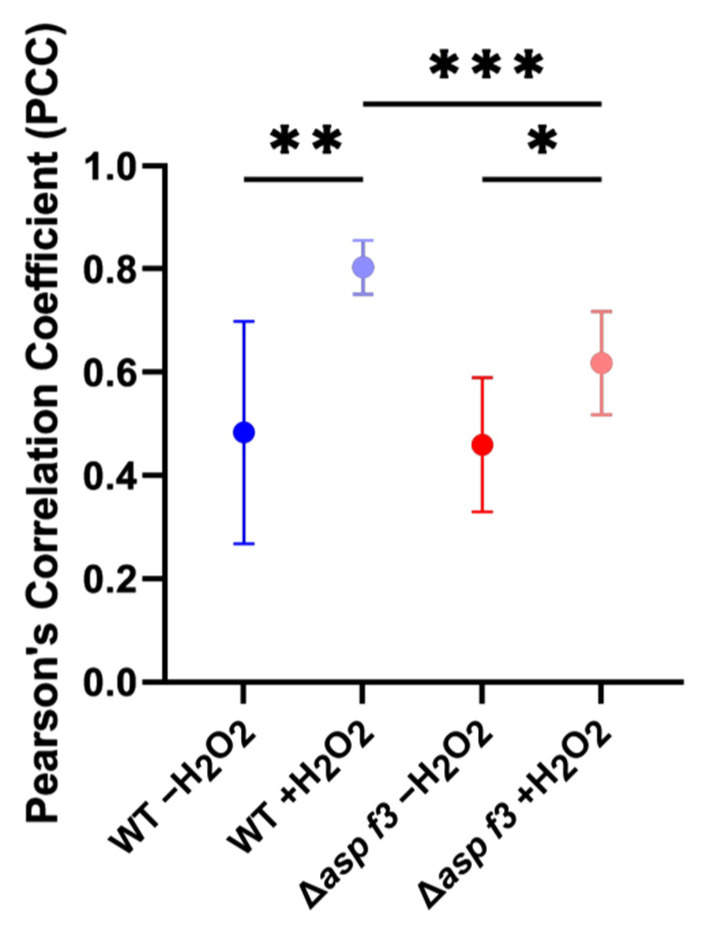
Quantification of co-localization of DAPI and AfYap1^VENUS^ dependent fluorescence signals. Pearson’s correlation coefficient (PCC) for DAPI and AfYap1^VENUS^ co-localization calculation in the presence and absence of H_2_O_2_ in the wild type and Δ*asp f3* background strains. The data represents the mean and standard deviation from at least three independent experiments (n ≥ 3). Significant differences calculated by Student’s t-test are shown as *: *p* < 0.05; **: *p* < 0.01; ***: *p* < 0.001.

The oxidative stress regulator Afyap1 localizes to the nucleus in response to ROS and activates the transcription of target genes, such as *cat2* [29]. The reduced expression levels of *cat2* in *Δasp f3*, as well as the lower catalase activity in the mutant, prompted us to evaluate whether Afyap1 activation depends on this peroxiredoxin. Thus, we monitored the subcellular localization of the AfYap1-Venus fusion protein in germlings of the wild type and Δ*asp f3* (Figure 6). In the absence of H_2_O_2_, AfYap1^VENUS^ displayed diffused cytosolic localization in both, wild type and Δ*asp f3* (0 min). An addition of 2 mM H_2_O_2_, induced nuclear localization of AfYap1^VENUS^ in wild type germlings within 30 min. Conversely, in the Δ*asp f3* background, a larger proportion of the AfYap1^VENUS^ remained diffused in the cytoplasm with only a minor nuclear concentration of the activator. To overcome heterogeneity in the microscopic data, we quantified the fluorescence intensity signals for AfYap1^VENUS^ and DAPI as a nucleus specific signal. Co-localization of the two different fluorescent intensities was determined as Pearson’s correlation coefficient (PCC, Figure 7). In the absence of H_2_O_2_, PCC values were 0.48 and 0.46 for wild type and Δ*asp f3*, respectively. Whereas, after 30 min of H_2_O_2_ exposure PCC values significantly increased for the wild type (0.80). However, a significantly lower PCC value was observed for Δ*asp f3* (0.61) when compared with the treated wild type indicating that efficient nuclear localization of Afyap1 depends on the peroxiredoxin Asp f3.

## 4. Discussion

The peroxiredoxin Asp f3 is a major allergen and an abundant protein of *A. fumigatus*, as an allergen on the conidial surface, but also within growing hyphae. It was shown to function in the defense against ROS and was essential during invasive aspergillosis in a mouse infection model [11]. However, it remained unclear how this confirmed biochemical function as a peroxiredoxin would serve precisely during infection in the host. In the absence of oxidative stress, the absence of Δ*asp f3* yields an inconspicuous phenotype, nearly indistinguishable from the wild type. Our transcriptome analysis confirmed, under in vitro conditions without ROS exposure, only minor transcriptional changes were detected and the Asp f3 protein appears to be dispensable for growth. This observation is in line with earlier results for *Saccharomyces cerevisiae* which demonstrated that the yeast was still viable despite a deletion of all five peroxiredoxin genes and that single mutants grew like the wild type in aerobic conditions [36]. Interestingly, the authors also showed that the Asp f3 orthologue in yeast, Tsa1p, secured long-term genomic stability by preventing mutations [37,38]. Such a protective function may well be conserved in *A. fumigatus* but would most likely not explain the avirulent phenotype of the Δ*asp f3* strain in the aspergillosis animal model.

While Asp f3 was dispensable in hyphae during the absence of ROS, confrontation with ROS induced major changes in the transcriptome. Resulting from its hypersensitive phenotype, one may have expected a slightly higher expression level of oxidative stress genes to compensate the phenotype, but indeed the opposite was observed. Several ROS defense genes were slightly downregulated under ambient growth conditions, and for others no induction was seen in response to ROS. Amongst the unaffected or even downregulated genes are several which are pivotal to the oxidative stress response or involved in virulence. Several of the genes, including *trxR*, *ccp1* and *gpx3*, also coincide with genes upregulated in *A. fumigatus* conidia when exposed to neutrophils, indicating their relevance during virulence [35]. Although not all of these gene products may be crucial to defend against innate immune cells, it confirms the presence of a perceptible exposure to ROS. Furthermore, TrxR was recently described as an essential gene which not only affects oxidative stress resistance but is needed for full virulence in animal models of both *Galleria melonella* and immunosuppressed mice [39]. Seven genes of the gliotoxin gene cluster are downregulated in Δ*asp f3*, including *gliP*, which encodes the nonribosomal peptide synthase catalysing the first step in the biosynthesis of gliotoxin. This mycotoxin is produced in vivo during infections and is known to mediate immunosuppressive effects on human cells [40,41,42,43]. When interacting with human neutrophils, gliotoxin was shown to re-organize the actin-skeleton, thus inhibiting phagocytosis and further inhibiting the respiratory burst and other neutrophil functions such as superoxide production [44,45]. Additionally, deletion of *gliP* was shown to attenuate virulence in mice immunosuppressed with hydrocortisone [42,46]. With regard to its reversible dithiol linkage, a regulatory link between gliotoxin biosynthesis and the fungal redox state seems likely and has previously been observed [47]. We saw a mild H_2_O_2_ dependent upregulation in the wild type and the opposite tendency in the absence of Asp f3. Such downregulation of the gliotoxin gene cluster in the Δ*asp f3* strain may lead to lower levels of the mycotoxin during infection which might thus attenuate its virulence potential in the lung environment in the host.

Some of the downregulated genes in Δ*asp f3* were confirmed regulatory targets of Afyap1. Furthermore, *Afyap1^VENUS^* overexpression in a Δ*asp f3* background could not compensate for the absence of Asp f3. These results suggested that Afyap1 and Asp f3 could be functionally interconnected, especially as a reversible disulphide bond formation is known to regulate Yap1 localization and activity. In baker’s yeast, activation of Yap1 was first reported to occur by the glutathione peroxidase (Gpx3), acting as the hydroperoxide receptor and redox transducer [48]. In our transcriptome the Gpx3 orthologue of *A. fumigatus* was clearly upregulated in the wild type in response to ROS but transcription remained unchanged in Δ*asp f3* after ROS treatment. It should not be excluded that lower levels of Gpx3 in the mutant may attenuate Afyap1 activation, either via direct interaction or as a member in a redox relay system. 

Peroxiredoxin dependent activation of the Yap1 regulator has also been proposed for filamentous fungi previously [49]. In *Aspergillus nidulans*, the Yap1 orthologue is coined NapA and regulates a wide set of genes far beyond ROS defense. Neither GpxA (Gpx3 in *A. fumigatus*) or two other peroxiredoxins, TpxA (AFUA_4g08580, Prx1 in *A. fumigatus*) and TpxB (AFUA_8G07130 in *A. fumigatus*), were found to be involved in NapA activation [50], making it seem unlikely that their orthologous proteins would fulfil this role in *A. fumigatus*. Both peroxiredoxins were slightly downregulated under oxidative stress, independent of Asp f3. Another peroxiredoxin, Tsa1p was shown to activate Yap1 in specific yeast strains [51]. Asp f3 is most likely not a true homologue of Tsa1p, as the amino acid identity between the two proteins is lower when comparing Asp f3 to Ahp1 (18% and 37%, respectively). In contrast to Tsa1p, Ahp1p is specific for alkylhydroperoxides [52].

We found Asp f3 to be the peroxiredoxin that mediates nuclear retention of Afyap1 under ROS exposure in *A. fumigatus*, indicating that this function may well be conserved for its homologue in *A. nidulans*-PrxA, which was found to be involved in oxidative stress defense and suspected to be the regulatory peroxiredoxin for NapA [49,53]. We speculate that this occurs via direct interaction of the two proteins and that rather its cellular abundance rather than specific biochemical properties of Asp f3 determine this interaction. As Afyap1 was previously found to be dispensable for virulence in a mouse model of aspergillosis our results also suggest that another, Afyap1 independent function of Asp f3 must be essential during infection conditions. A previous study has identified that iron availability may be compromised in response to oxidative stress [54] and Asp f3 may represent a regulatory hub between these interconnected stress responses. 

## 5. Conclusions

*Aspergillus fumigatus* puts immunocompromised patients at a high risk of severe and often fatal infections. It is thus imperative to find not only more reliable diagnostic tools but also research a more targeted approach for antimycotic treatment. In this study we investigated the in vivo function of Asp f3, a protein that plays an essential in virulence. A transcriptomic approach showed clear differences between the wild type and the highly ROS-sensitive Δ*asp* f3 mutants. Further investigations led to the conclusion that Asp f3 deficient mutants suffer from a deregulation of the oxidative stress response due to lacking nuclear retention of the regulator Afyap1. However, loss of Afyap1 does not lead to avirulence of *A. fumigatus*, strongly suggesting additional cellular effects upon challenge with ROS.

## Figures and Tables

**Table 1:** Strains of *Aspergillus fumigatus*.

Strain	Genotype	References
*A. fumigatus* D141	WT	[15]
*A. fumigatus* Δ*asp f3*	Asp f3::hph; Hyg^R^	[11]
*A. fumigatus* Δ*asp f3^C^*	Asp f3::hph; Hyg^R^ Δasp f3::Asp f3; PT^R^	[11]
*A. fumigatus* OE::*Afyap1^VENUS^*	P_GpdA_-Afyap1^Venus^-T_nos_::ptrA; PT^R^	This study
*A. fumigatus* Δ*asp f3* OE::*Afyap1^VENUS^*	Asp f3::hph; Hyg^R^ P_GpdA_-Afyap1^Venus^-T_nos_::ptrA; PT^R^	This study

**Table 2:** Expression of oxidative stress genes in the *Aspergillus fumigatus* wild type and Δ*asp f3* in response to reactive oxygen species (ROS).

Gene ID		Afyap1 Target *	WT + ROS vs. WT − ROS	Δ*asp f3* + ROS vs. Δ*asp f3* − ROS	Δ*asp f3* + ROS vs. WT + ROS
1	Putative NADH flavin oxidoreductase (AFUA_2g04060)	−	4.16	1.93	−2.65
2	bifunctional catalase-peroxidase (*cat2*, AFUA_8g01670)	+	2.85	0.31	−2.64
3	p-Nitroreductase family protein (*pnr1*, AFUA_5g09910)	+	4.05	2.4	−2.15
4	Oxidoreductase, putative (AFUA_5G01250)	−	2.12	0.39	−1.87
5	Thioredoxin reductase (*trxR,* AFUA_4g12990)	−	2.75	1.79	−1.74
6	Glutathione transferase, putative (AFUA_2g15770)	−	2.53	0.95	−1.66
7	NADH-dependent flavin oxidoreductase, putative (AFUA_7G06420)	−	1.81	0.88	−1.47
8	Glutathione peroxidase (*gpx3,* AFUA_3g12270)	−	1.31	0.35	−1.34
9	Cytochrome c peroxidase (*ccp1*, AFUA_4G09110)	+	1.36	0.21	−1.3
10	Glutathione S-transferase, putative (AFUA_2G00590)	−	0.85	0.22	−1.18
11	Ferric-chelate reductase, putative (AFUA_6G13750)	−	0.86	−0.26	−1.14
12	Gliotoxin Cluster e.G. gliM (AFUA_6G09680)	−	−0.3	0.62	−1.12
13	Metalloreductase, putative (AFUA_6g02820)	−	1.21	0.27	−1.1
14	Thioredoxin (Asp29/Trx1) (AFUA_5g11320)	−	1.63	0.7	−1.1
15	Mitochondrial peroxiredoxin Prx1 (AFUA_4G08580)	+	−0.75	−0.66	0.13
16	Methionine synthase MetH/D (AFUA_4G07360)	+	−0.11	−0.02	0.07

* Putative members of the Yap1 regulon according to Lessing et al., 2007 [29].

## Data Availability

The sequencing data have been deposited in NCBI’s Gene Expression Omnibus database and are accessible through GEO Series accession number GSE173349 (https://www.ncbi.nlm.nih.gov/geo/query/acc.cgi?acc=GSE173349, accessed on 28 February 2021).

## Supplementary Materials

The following are available online at https://www.mdpi.com/article/10.3390/genes12050668/s1, Figure S1: Overexpression construct for Afyap1^VENUS^. A Vector Map of gpdA-*Afyap1-VENUS* including the pyrithiamine resistance gene *thiA (ptrA)* gene of *Aspergillus oryzae* as a selectable marker for successful transformation in *A. fumigatus*. Figure S2: Growth of *Aspergillus fumigatus* on minimal media (AMM) with various carbon sources. 2 × 10^4^ conidia of the wild type (D141) and the *asp f3* deletion mutant (Δ*asp f3*) were point-inoculated on AMM with indicated supplements (% *w*/*v*) as carbon/nutrient sources and incubated at 37 °C for 48 h. The *Afyap1* deletion strain (Δ*Afyap1*) and its wild type like parent CEA17Δ*aku^BKU80^* (CEA17) are shown for comparison. Table S1: Primer list for the generation of *Afyap1*^VENUS^. Table S2: Results of the gene enrichment analysis (GO-term search, molecular function). Table S3: Results of the gene enrichment analysis (GO-term search, biological process).
