# Supplementary material for: The Peroxiredoxin Asp f3 Acts as Redox Sensor in Aspergillus fumigatus
Source: Genes (Basel). 2021 Apr 29;12(5):668. doi: 10.3390/genes12050668 (PMC8145481; doi:10.3390/genes12050668)
Supplement: Supplementary file 1 [file genes-12-00668-s001.zip › genes-1166454-supplementary.pdf]

Supplementary Information - Boysen et al.,

Supplementary Figures

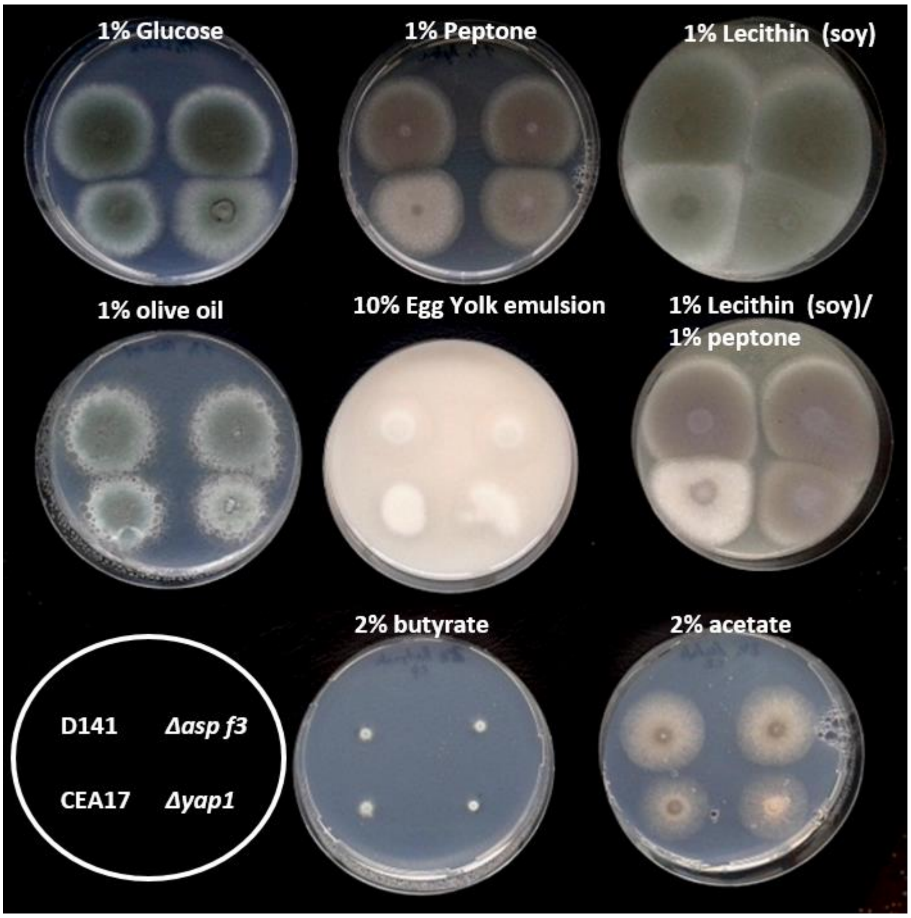

Supplementary Fig. 1: Growth of *Aspergillus fumigatus* on minimal media (AMM) with various carbon sources. 2×10^4 conidia of the wild type (D141) and the *asp f3* deletion mutant (*ΔaspF3*) were point-inoculated on AMM with indicated supplements (% w/v) as carbon/nutrient sources and incubated at 37°C for 48 h. The *Afyap1* deletion strain (*Δyap1)* and its wild type like parent CEA17ΔakuB^{KU80} (CEA17) are shown for comparison.

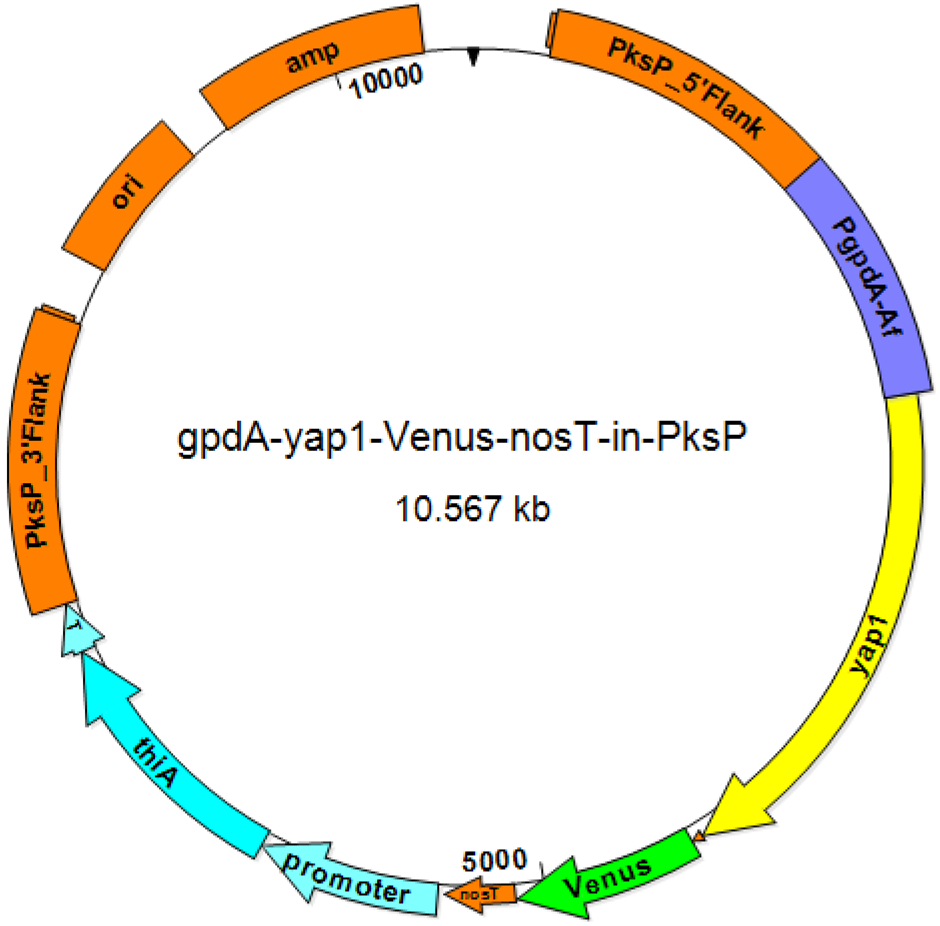

Supplementary Fig. 2: Overexpression construct for *Afyap1*^{VENUS}. **A** Vector Map of gpdA-*Afyap1-VENUS* including the pyrithiamine resistance gene *thiA* (*ptrA*) gene of *Aspergillus oryzae* as a selectable marker for successful transformation in *A. fumigatus*.

Supplementary Table 1: Results of the gene enrichment analysis (GO-term search, molecular function)

GO name	GO ID	Gene ID
oxidoreductase activity	GO:0016491	AFUA_1G02820; AFUA_1G03250; AFUA_1G12460; AFUA_1G14520; AFUA_1G14540; AFUA_1G15610; AFUA_2G00170; AFUA_2G00460; AFUA_2G04060; AFUA_2G04330; AFUA_2G06000; AFUA_2G09580; AFUA_2G16570; AFUA_2G17850; AFUA_3G00180; AFUA_3G00865; AFUA_3G01290; AFUA_3G01780; AFUA_3G03030; AFUA_3G09540; AFUA_3G12270; AFUA_3G15350; AFUA_4G09920; AFUA_4G12990; AFUA_4G14490; AFUA_5G01030; AFUA_5G01250; AFUA_5G01290; AFUA_5G01450; AFUA_5G02020; AFUA_5G07000; AFUA_5G08900; AFUA_5G09910; AFUA_5G10060; AFUA_5G10070; AFUA_5G14000; AFUA_5G14800; AFUA_6G00280; AFUA_6G02820; AFUA_6G09670; AFUA_6G10120; AFUA_6G10720; AFUA_6G13750; AFUA_7G06260; AFUA_7G06420; AFUA_7G06600; AFUA_8G01550; AFUA_8G01630; AFUA_4G09110; AFUA_8G01670; AFUA_6G02280; AFUA_4G10770;
inorganic phosphate transmembrane transporter activity	GO:0005315	AFUA_3G03010; AFUA_7G06350; AFUA_8G01850;
peroxidase activity	GO:0004601	AFUA_3G12270; AFUA_4G09110; AFUA_8G01670; AFUA_6G02280; AFUA_4G10770;
glutathione transferase activity	GO:0004364	AFUA_2G00590; AFUA_3G10830 (GstA); AFUA_6G09690;
FMN binding	GO:0010181	AFUA_1G02820; AFUA_2G04060; AFUA_3G00865; AFUA_5G01450 AFUA_7G06420;

Supplementary Table 2: Results of the gene enrichment analysis (GO-term search, biological process)

GO name	GO ID	Gene ID
Oxidation-reduction process	GO:0055114	AFUA_1G02820 (*Pst2*); AFUA_1G03250; AFUA_1G12460; AFUA_1G14520; AFUA_1G14540; AFUA_1G15610; AFUA_2G00170; AFUA_2G00460; AFUA_2G04060; AFUA_2G04330; AFUA_2G06000; AFUA_2G09580; AFUA_2G16570; AFUA_2G17850; AFUA_3G00180; AFUA_3G00865; AFUA_3G01290; AFUA_3G01780; AFUA_3G02300; AFUA_3G03030; AFUA_3G09540; AFUA_3G10830 (*GstA*); AFUA_3G12270; AFUA_3G15050; AFUA_3G15350; AFUA_4G09920; AFUA_4G12990 (trr1); AFUA_4G14490; AFUA_5G01030; AFUA_5G01250; AFUA_5G01290; AFUA_5G01450; AFUA_5G02020; AFUA_5G07000; AFUA_5G08900; AFUA_5G09910; AFUA_5G10060; AFUA_5G10070; AFUA_5G11320; AFUA_5G14000; AFUA_5G14800; AFUA_6G00280; AFUA_6G02820; AFUA_6G09670 (*GliC*); AFUA_6G09730 (*GliF*); AFUA_6G10120 (*ToxD*); AFUA_6G10720; AFUA_6G13750; AFUA_7G06260; AFUA_7G06420; AFUA_7G06600; AFUA_8G01550; AFUA_8G01630; AFUA_4G09110 (*ccp1*); AFUA_8G01670 (*Cat2*); AFUA_6G02280 (*asp f3*);
mycotoxin biosynthetic process	GO:0043386	AFUA_6G09660 (*GliP*); AFUA_6G09670 (*GliC*), AFUA_6G09680 (*GliM*), AFUA_6G09690 (*GliG*), AFUA_6G09710 (*GliA*), AFUA_6G09720 (*GliN*), AFUA_6G09730 (*GliF*),
transmembrane transport	GO:0055085	AFUA_1G10390; AFUA_1G12620; AFUA_1G13350; AFUA_1G13360; AFUA_1G17160; AFUA_2G02040; AFUA_2G05840; AFUA_2G16860; AFUA_3G03010; AFUA_3G11490; AFUA_3G12010; AFUA_3G14560; AFUA_3G15250; AFUA_4G00120; AFUA_4G01050; AFUA_4G12260; AFUA_4G13820; AFUA_4G13830; AFUA_5G00790; AFUA_5G07970; AFUA_5G11980; AFUA_6G03860; AFUA_6G04360; AFUA_6G09710 (GliA); AFUA_6G12550; AFUA_7G00390; AFUA_7G05100; AFUA_7G06350; AFUA_7G06390; AFUA_8G00770; AFUA_8G01850; AFUA_8G04470; AFUA_8G06610
phosphate ion transport	GO:0006817	AFUA_3G03010, AFUA_7G06350, AFUA_8G01850,
response to oxidative stress	GO:0006979	AFUA_3G12270, AFUA_4G09110 (ccp1), AFUA_8G01670 (Cat2), AFUA_4G10770,

Supplementary Table 3: Primer list for the generation of *Afyap1*^{VENUS}

Primer	Sequence	Application
PksP_5'F_OH_BB_F	GCCTCTTCGCTATTACGCCAGCAATTCGAGGATGTCTTCCG	Amplification of PksP 5'Flank
PksP_5'F_OH_gpdA_R	CGGAACAATGTTAATCCACAAACTACGGAGTAGTGATTATCCTGGACC	Amplification of PksP 5'Flank
PksP_3'F_OH_Pth_F_neu	GGGGCATTCTAGAATAATTATGTGTAACAAGAAAGAGGGGTGGAGTTGTGCATACC	Amplification of PksP 3'Flank
PksP_3'F_OH_BB_R_neu	GTTGGCCGATTCATTAATGCAGTTCATTGGGGGCGGTTACTCG	Amplification of PksP 3'Flank
PksP_5'F_F	CAATTCGAGGATGTCTTCCG	Generation of Afyap1-VENUS overexpression fragment
PksP_3'F_R	GGCTTTGTCCAATCACATCCAGC	Generation of Afyap1-VENUS overexpression fragment
PgpdA_F_1	GTAGTTTGTGGATTAACATTGTTCCG	Amplification of *gpdA*-promoter sequence
P_gpdA_rev	TGTGTAGATTCGTCTGGTACTGAGC	Amplification of *gpdA*-promoter sequence
Yap1_F	ATGGCGGACTACAATACTC	Amplification of Afyap1-Gene
Yap1_OH_Link_V_R	CCTCCGCCAGATCCGCCGCCTCCTTTGACGCGACCCATGATGTCC	Amplification of Afyap1-Gene
pUC_BB_fw	CTGCATTAATGAATCGGCCAACGC	Generation of pUC-vector backbone
pUC_BB_rev	CTGGCGTAATAGCGAAGAGGC	Generation of pUC-vector backbone
VENUS_Link_Fwd	GGAGGCGGCGGATCTGGCGGAGG	Generation of combined Linker-VENUS and pyrithiamine resistance cassette
Pth fwd	TCTTTCTTGTTACACATAATTATTCTAGAATGCCCC	Generation of combined Linker-VENUS and pyrithiamine resistance cassette
